# Killer Yeasts for the Biological Control of Postharvest Fungal Crop Diseases

**DOI:** 10.3390/microorganisms8111680

**Published:** 2020-10-29

**Authors:** Mariana Andrea Díaz, Martina María Pereyra, Ernesto Picón-Montenegro, Friedhelm Meinhardt, Julián Rafael Dib

**Affiliations:** 1 Planta Piloto de Procesos Industriales Microbiológicos–CONICET, Av. Belgrano y Pje. Caseros, Tucumán 4000, Argentina; dmarianaandrea@gmail.com (M.A.D.); martinapereyra30@gmail.com (M.M.P.); epiconm99@gmail.com (E.P.-M.); 2 Institut für Molekulare Mikrobiologie und Biotechnologie, Westfälische Wilhelms Universität Münster, Corrensstr. 3, 48149 Münster, Germany; 3 Instituto de Microbiología, Facultad de Bioquímica, Química y Farmacia, Universidad Nacional de Tucumán, Ayacucho 471, Tucumán 4000, Argentina

**Keywords:** killer yeast, biological control, crop diseases, fruit, postharvest, preharvest

## Abstract

Every year and all over the world the fungal decay of fresh fruit and vegetables frequently generates substantial economic losses. Synthetic fungicides, traditionally used to efficiently combat the putrefactive agents, emerged, however, as the cause of environmental and human health issues. Given the need to seek for alternatives, several biological approaches were followed, among which those with killer yeasts stand out. Here, after the elaboration of the complex of problems, we explain the hitherto known yeast killer mechanisms and present the implementation of yeasts displaying such phenotype in biocontrol strategies for pre- or postharvest treatments to be aimed at combating postharvest fungal decay in numerous agricultural products.

## 1. Introduction

Postharvest fungal diseases of fruit and vegetables cause major crop losses ranging from 25% in industrialized up to 50% in developing countries [1]. Counteractive measures include chemical, physical, and biological approaches. Fungicides most frequently serve to combat fungal infestations of field crops as such chemically synthesized compounds are rather inexpensive, can be stored for long periods of time, and they are sufficiently effective. However, concern is continuously rising over their routine use because, besides threatening human health [2,3,4] and the environment [5], resistant fungal pathogenic biotypes concomitantly arose [6,7,8]. It is, thus, not surprising that a number of countries, such as the USA and those that form the European Union, promote a project called “Integrated Pest Management (IPM)” which aims at reducing or, whenever possible, completely replacing chemical pesticides [9,10,11,12,13,14].

## 2. Counteractive Measures to Control Fungal Diseases

### 2.1. Preharvest Stage

Since prevention is better than cure, postharvest disease control should start at best prior to the actual harvesting process, not least owing to the fact that numerous potential pathogens are capable of performing a latent infection. Resting in the peel, germs start to thrive and prosper when the fruit—during harvesting—becomes wounded [15]. In fact, preharvest measures to be aimed at preventing crop losses are frequently applied, as for citrus, for which copper compounds are routinely deployed to combat the brown rot caused by *Phythophthora citrophthora* [16]. For subtropical fruit—among them mangoes, papaya, and bananas—protective fungicide sprays are systematically used to battle against the anthracnose disease elicited by *Colletotrichum gloeosporioides* [17]. There is, however, cause for concern due to the nascency of resistant strains; thus, stingy employments of fungicides are imperative [18].

### 2.2. Postharvest Stage

Mechanic handling is an indispensable and clearly fruit-sanitary relevant factor as it affects peel integrity [19,20]. Besides controlling mechanical damage to minimize the entry of potential pathogens [19,20], various alternative postharvest measures (physical, chemical and biological or combinations thereof) were applied to ward off fungal infestations [18].

High or low temperatures and gamma irradiation are among the most frequently applied postharvest physical treatments to reduce the pathogen´s population on fruit surfaces. There is, however, a major inherent disadvantage as such strategies do not cause long-lasting protective effects, as seen, e.g., for chemical agents [18]. Hence, chemical pesticides are widely applied to control crop diseases, but their use is fraught with risk, and that is why the public opinion as well as the academic community is skeptical of their usage. Strong doubts exist mainly due to their toxicity, environmental pollution, and the emergence of resistant strains. GRAS (Generally Recognized As Safe) compounds may serve as alternatives as they a priori do not threaten human health or the environment. Indeed, several salts and other ecologically harmless substances displayed control effects, and they have in fact been frequently used in lab tests, often along with biological agents; e.g., *Cryptococcus laurentii* (*Kufferath*) combined with 2% of sodium bicarbonate was successfully applied to control *Penicillium digitatum* in oranges [21]. Likewise, *Pichia guilliermondii* along with CaCl_2_ served to fight *P. digitatum* in grapefruits [22], and *Candida oleophila* together with Mg^2+^ and Ca^2+^ salts were shown to control *Botrytis cinerea* and *Penicillium expansum* in in vitro assays [23].

### 2.3. Biological Control

A number of microorganisms able to protect fruit from fungal infections have been identified, including bacteria, filamentous fungi and yeasts [24,25]. The latter are clearly advantageous over the others as their nutritional requirements are rather simple, they can easily be produced in high yields with inexpensive substrates, they are not harmful to humans, the environment or host fruit, and—not least—the target organisms are unlikely to generate resistance [26].

In addition to the search for microbial antagonists, emphasis was put on understanding possible action mechanisms because such knowledge most probably offers opportunities to improve the protective effectiveness. Among the several identified modes of action, the most common mechanism simply concerns competition for space and nutrients [27,28,29]. However, there are other types, such as direct parasitism [30,31], production of siderophores, antibiotics and volatile compounds [32,33,34,35], and those modes of actions subsumed under the term killer phenotype [28].

## 3. Yeasts as Biocontrol Agents

Besides rather sophisticated mechanisms enabling yeasts to restrict or kill competitors (see the following chapter), there are some general characteristics qualifying these unicellular eukaryotes (in its broadest sense) for protection: they use nutrients rapidly and proliferate abundantly; they have the ability to colonize fruit surfaces displaying rather long immobilization times enduring even dry conditions; they produce extra-cellular polysaccharides enhancing their own survival, concomitantly restricting pathogens; and they sufficiently tolerate commonly applied pesticides, such as imazalil, tiabendazole, pyrimethanil or fludioxonil [36,37,38,39,40].

Though numerous reports regarding the use of yeasts as biological control agents exist, the number of commercial products is limited and, indeed, covers the potential market only at a small fraction. Multiple and time-consuming steps are most critical for product development and implementation, and that is why the involvement of a commercial company with expertise in upscaling, formulation, registration procedures, and the marketing network is a must to successfully meet above challenges [41].

**Figure 1:**
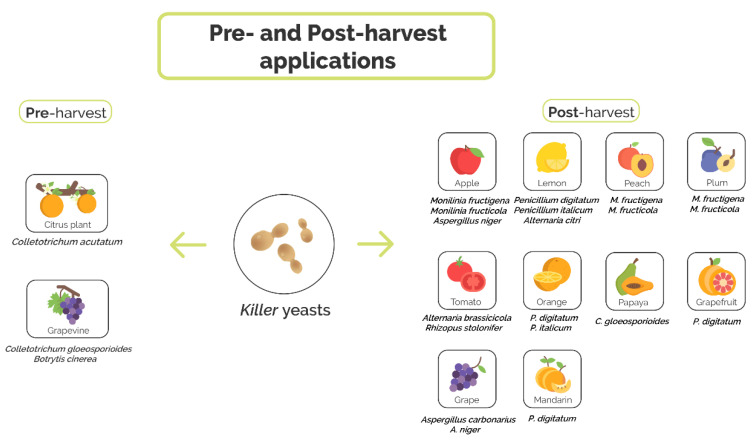
Applications of killer yeasts in agriculture to prevent fungal infections. Killer yeasts have been used for both pre- and postharvest treatments.

Reviews about killer yeasts and their applications in medicine, industry and agriculture have been written over the years [42,43,44,45]. Here, we focus on representatives which actually function or presumably can act as candidates for antagonizing harmful –most commonly filamentous– fungi known to cause postharvest rotting of edible fruit and vegetables. After dealing with killer yeasts in general, we present an up-to-date compilation of the literature with respect to the aforesaid application which is schematically outlined in Figure 1.

### 3.1. The Yeast Killer Phenotype

Killer substances are produced by pro- and eukaryotic cells [44,46,47,48,49]. They clearly improve the organisms’ capability to dominate a certain environmental niche by killing or inhibiting competing microorganisms. However, the killer phenotype is not always implemented solely for being advantageous for its host as the primary function rather often apparently ensures auto-selection of the extrachromosomal elements encoding the killer toxin (virus-like particles (VLPs) or virus-like elements (VLEs)) since it eliminates any cell that does not or no longer carries the cytoplasmic VLP or VLE [50]. Nevertheless, as a collateral action it is advantageous for the producing cell as it inhibits competing organisms.

The killer phenotype was first described for the baker’s yeast *Saccharomyces cerevisiae* by Bevan and Makower in the year 1963 [51]. They differentiated three phenotypes, i.e., killer, sensitive and neutral: a killer yeast is capable of producing toxins that cause the death of sensitive yeasts, a neutral yeast is not a toxin producer and is also not affected by the toxin. Woods and Bevan [52] realized the proteinaceous nature of the toxin, and they checked optimal conditions for its action: such as low pH, chemical composition of the growth medium, and the physiological state of the cells affecting the amount of the killing toxin. Initially, it was suggested that killer yeasts are lethal only to other yeasts, but further studies evidenced that they can be equally toxic to other fungi or even bacteria [53,54].

### 3.2. Genetics of the Killer Phenotype

First studies on the nature of the killer factor revealed the involvement of cytoplasmic genetic determinants [55]. Double stranded RNA (dsRNA) associated with VLPs was proposed and approved as the genetic basis [52,55,56,57,58,59,60]. Today, killer phenomena are known for a large number of yeasts [61,62]. Toxins cannot only be extra-chromosomally encoded but also chromosomally [44].

**Figure 2:**
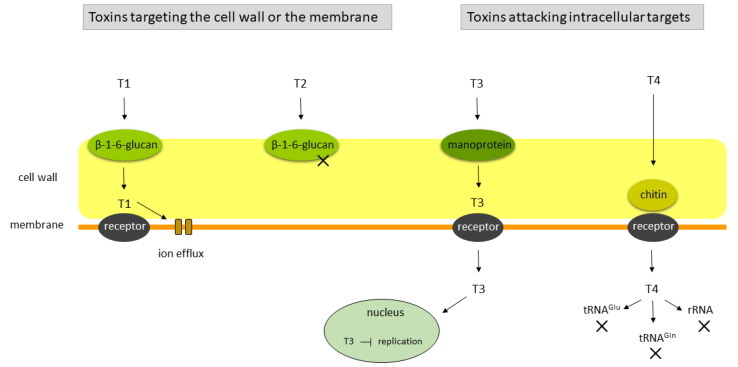
Modes of action of killer toxins according to Klassen et al. (2017), modified [61]. Killer toxins can be divided into four categories regarding the cell target. The T1 and T2 toxin types use β-1,6-glucan as the primary cell wall receptor (in figure, toxins targeting the cell wall or the membrane). T1-type toxins such as K1, K2 and PMKT [65,66,67,68,69] bind to a membrane receptor and induce membrane pore formation; T2-type toxins, widely recognized in the genus *Wickerhamomyces* [70], act as glucanases. T3 and T4 toxin types have intracellular targets. K28 encoded by *S. cerevisiae* [47,63] is an example of a T3 toxin, which uses mannoproteins as the primary receptor, reaches the nucleus via retrograde transport and inhibits DNA synthesis. T4-type toxins, such as zymocin, PaT and PiT [64,71,72,73] first bind to a chitin receptor in the cell wall. Subsequently, a membrane receptor is required for the cellular uptake of the toxic subunit which cleaves the respective RNA target (tRNAglu, tRNAgln and rRNA).

Killer toxins are often classified with respect to their cellular target. Essentially, there are four categories: (a) toxins capable of generating membrane pores followed by cellular lysis; (b) toxins displaying glucanase activity, eventually resulting in cell wall destruction and target cell lysis; (c) toxins disturbing nuclear functions by blocking replication; and (d) toxins targeting cellular RNAs such as specific tRNAs or rRNA (see also Figure 2).

For more detailed information on killer toxin action and self-protection of killer yeasts, we refer to recent reviews and publications dealing with the specific subject in depth: Schaffrath et al. (2018) [44]; Schmitt and Breining (2002, 2006) [47,63]; Kast et al. (2015) [64]; Satwika et al. (2012) [50].

## 4. Application of Killer Yeasts as Biocontrol Agents

Fruit and vegetables seem to provide a favorable environment for yeasts in general and for killer yeasts in particular since approximately a quarter of the strains isolated from such source display the phenotype [42,45]. Killer yeasts have a great potential to act as biocontrol agents, producing toxins which do not harm humans or the fruit [74]. Moreover, killer yeasts display fruit-protective qualities in both pre- and postharvest applications.

### 4.1. Preharvest Application of Killer Yeast

Though there is only a limited number of studies dealing with killer yeasts as preharvest antagonists, such as in citrus and grapes, the reported cases clearly furnish evidence of their biocontrol potential when applied this way.

A rather common preharvest fungal pathogen of grapes is *C. gloeosporioides* which generates an anthracnose disease characterized by irregular-shaped black necrotic spots, the centers of which—in the course of the disease—become whitish gray surrounded by narrow reddish-brown to black margins [75]. For controlling anthracnose, grapes are routinely medicated with fungicides such as fluazinam and chlorothalonil [76].

Only rather recently, three killer yeasts were isolated displaying efficient antagonistic activity against the anthracnose causative pathogen. The protective potential of *S. cerevisiae* GA8, *S. cerevisiae* LFA802 and *S. cerevisiae* L24 was proven. Each of the three strains was able to produce extracellular antifungal agents such as β-1, 3-glucanases and chitinases; strain GA8 additionally synthesized volatile compounds and thermostable metabolites with the potential to obstruct *C. gloeosporioides*. In addition, such strains were able to colonize wounds and stably maintained a vital state on the surface of the berries. Moreover, each of them was able to inhibit germination of the phytopathogen’s spores [77].

*B. cinerea* is a wide spread, ubiquitously occurring fungus; it is the causative agent of the gray mold disease in a wide range of hosts provoking considerable yield losses of grape, lettuce, onion, potato, strawberry, tomato, and other fruits of commercial interest. In 2004, Santos et al. [78] purified and characterized the CYC 1106 killer toxin from *Pichia membranifaciens*, a strain isolated from olive brines to be used against grey mold. In in vivo assays, *Vitis vinifera* plants developed the characteristic grey mold symptoms when inoculated with *B. cinerea* (control treatment). However, all of the plants treated with a mixture of *P. membranifaciens* and *B. cinerea* remained healthy, while 20% of the plants treated solely with the purified toxin depicted the pathology. According to the authors, such differences could be attributed to other yeast action mechanisms such as the production of hydrolytic enzymes or the competition for nutrients [78].

*Colletotrichum acutatum* is one of the most devastating fungi affecting citrus during preharvest stages. It is the causative agent of the post bloom fruit drop. By attacking flower tissues and changing normal fruit abscission persistent calyces and peduncles develop. Lopes et al. (2015) [79], investigated six *S. cerevisiae* killer yeast strains (ACB-CR1, ACB-KD1, ACB-CAT1, ACB-BG1, ACB-K1 and ACB-PE2) isolated from commercial ethanol fermentations in Brazil with respect to their bio-control potential for *C. acutatum*. ACB-CAT1 and ACB-CR1 in vitro most efficiently inhibited mycelial growth, i.e., 71% and 67%, respectively. All of the isolates were both, curative and preventive: 73–84% of the flowers were asymptomatic after curative treatments, and 50–86% remained healthy when the treatment was preventive. After analyzing putative action mechanisms, all strains were shown to produce antifungal compounds; they competed for nutrients, inhibited the pathogen´s spore germination, and produced killer toxins and hydrolytic enzymes.

### 4.2. Postharvest Application of Killer Yeasts

In spite of the rather promising results obtained in preharvest biocontrol attempts, postharvest use of killer yeasts is most common [80].

**Figure 3:**
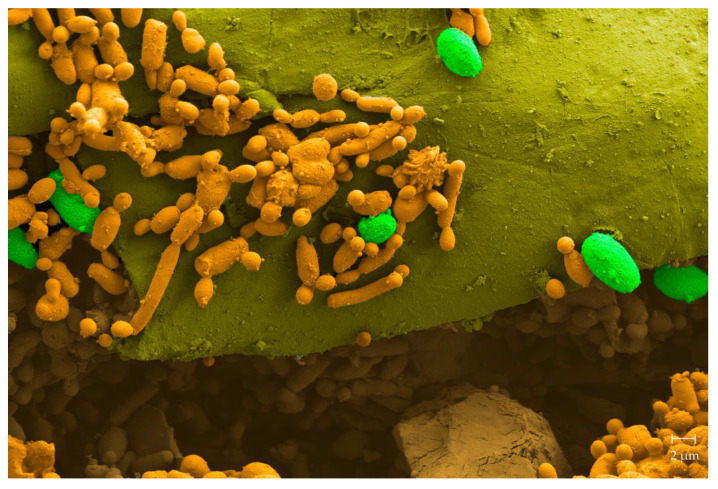
Colored scanning electron microscope (SEM) image of the surface of a lemon wound inoculated with the killer yeast *C. lusitaniae* 146. The image shows a cross section of a lemon wound where colonization by the killer yeast (orange) and ungerminated spores of citrus phypathogen *P. digitatum* (green) are observed.

In citrus, the main fungal postharvest affliction is the “green mold disease” produced by *P. digitatum*. Control efforts routinely include chemical fungicides such as imazalil or thiabendazole [81]. Seeking for safer alternatives, we isolated more than 400 native yeasts originating from citrus, 8.5% of which showed killer activities [82]. In in vivo assays, two strains of *Pichia* sp. (strains 27 and 28), and *Wickerhamomyces* sp. strain 56 conferred significant shelter from the decay caused by *P. digitatum*. Efficiencies reached 93.6%, 82.5%, and 72.5%, respectively. Furthermore, the native killer yeast *Clavispora lusitaniae* 146 displayed, again in in vivo tests, strong antagonistic activities against the fungal pathogen (Figure 3), conferring consistent protection during the entire harvesting period [39]. The biocontrol potential of *C. lusitaniae* 146 was seen to be stronger than that of a commercial product containing *C. oleophila*. Moreover, the strain was able to grow with agriculturally applied concentrations of fungicides, which allows for the combined use of the yeast along with—then minor—doses of the fungicides. The strain’s commercial potential unequivocally became evident when we studied action mechanisms, such as wound colonization and the inhibition of *P. digitatum* spore germination, as *C. lusitaniae* 146 was seen to efficiently control even a fungicide-resistant *P. digitatum* strain. In addition, strain 146 recently demonstrated a broad range of protective activity in various citrus varieties besides lemons, such as mandarins, oranges, and grapefruits [83,84,85].

Several killer species are able to control the green mold disease of the Tarocco orange (*Citrus sinensis*), a predominant cultivar in Eastern Sicily/Italy [86]. *S. cerevisiae* (strains BS46 and BCA61) and *Wickerhamomyces anomalus* (strains BCU24, BS91, BS92, and BCA15) isolated from fermented olives were tested in vivo and in vitro for their capability to restrain *P. digitatum*. Only *W. anomalus* gave positive results in vitro; the in vivo antagonism assays eventually identified *W. anomalus* BS91, BS92 and BCA15 to be most effective with respect to the reduction of the disease incidence (1, 4 and 44%, respectively). The deleterious and vice versa the positive effect on the pathogen and the cultivar, respectively, lasted up to 10 storage days counted from the beginning of the artificially provoked infection. In vitro, *W. anomalus* BS91 had an effect on mycelial structures of *P. digitatum*: the hyphae became wilted, folded, and coiled with a grainy appearance, an effect presumably due to the production of hydrolytic enzymes by BS91 (β-1, 3-glucanase) as previously reported by Muccilli et al. [70,87].

Only rather recently yeasts capable of antagonizing *Penicillium italicum* in “Valencia” sweet oranges ((*Citrus sinensis* (*L.*) *Osbeck* (*rutaceae*)) were isolated [88]. The fungus can infect injured fruit peels, generating the symptomatology known as “blue mold disease”. Sapping all parts of the fruit the fungus makes infected oranges uneatable. From 14 citrus producing areas of the São Paulo State/Brazil, ninety-seven yeast strains were isolated from leaves, flowers, fruits, and soils. Screening for protective representatives was performed in vivo and in vitro. *Candida stellimalicola* strains ACBL-04 and ACBL-05, and *S. cerevisiae* ACBL-11 and ACBL-10 were most successful in preventive treatments, whereas ACBL-08 additionally displayed curative effects. With respect to the control mechanism, the killer trait appeared to be crucial for the biocontrol of *P. italicum*.

*Monilinia fructigena* and *Monilinia fructicola* are major pathogens of stone fruit (peaches and plums). They cause a panoply of symptoms including blossom blighting, woody cankers, and preharvest fruit rotting; however, the most serious damage happens after harvesting during transport and storage. Since currently there is no efficient treatment to control *Monilinia* spp. infections, biocontrol agents, among them killer yeasts, were considered to presumably meet the challenge [89]. *Debaryomyces hansenii* MI1a and *D. hansenii* K12a, isolated from blue-veined Rokpol cheese, and *W. anomalus* BS91, previously shown control *P. digitatum* in oranges (see above and [86]), were selected due to their killer phenotype and antagonistic activity against other fungal pathogens. Exclusively *W. anomalus* BS91 was able to inhibit both of the *Monilinia* pathogens by producing volatile antifungal compounds in an in vitro dual culture method. In vivo antagonism tests identified *D. hansenii* MI1A as totally *M. fructicola* ineffective in both plum and peach, and there was only a weak impact on *M. fructigena*. *D. hansenii* K12a and *W. anomalus* BS91 conferred clear fruit protection. When peaches were treated 24 h prior to inoculation with *M. fructigena*, the disease severity was mitigated to 33 and 25%, respectively. For the plums, a similar effect against *M. fructigena* was seen. The effect observed for peaches was even more promising as both, *D. hansenii* K12a and *W. anomalus* BS91 lessened disease severity caused by *M. fructicola* to 55%, when applied 24 h before the fungus was added. For plums, the best results were obtained with *W. anomalus* BS91 that reduced disease severity to 30%.

Cells and killer toxins from *D. hansenii* strains (TEM8 and TEM7), previously isolated from a Turkish-style homemade dairy product [90], were tested for their biocontrol efficiency in tomato against *Alternaria brassicicola* and *Rhizopus stolonifer* [91]. *A. brassicicola* is a necrotrophic fungus that causes the black spot disease in a broad range of host plants [92] and *R. stolonifer* is an important soft rot-causing fungus affecting tomatoes during postharvest stages [93]. The efficiency of the toxin was compared with the action of viable yeast cells. When the fruit was inoculated with a killer yeast or treated with killer toxins prior to applying fungal spore suspensions, the lesion sizes were smaller than for the control, and there were even samples with no damages at all. Furthermore, the killer proteins and the cells also cropped up to be effective against *Alternaria citri* and *A. niger* in lemons and apples, indicating a wider application spectrum than solely for tomatoes.

In the north of Brazil where papaya crops agriculturally predominate, *C. gleosporoides*, the causative agent of anthracnosis, commonly produces severe postharvest losses. Additionally, the pathogen effectuates small reddish-brown surface patches unattractively modifying the fruit´s physical appearance. Two killer yeast species isolated from street markets and producer farms in the Ceará State/Brazil, *W. anomalus* 422 and *Meyerozyma guilliermondii* 443 [94] were able to reduce the diameter of disease lesions by 31.4 and 41.17%, respectively, when applied 24 h prior to the application of the pathogen. Such killer yeasts displayed protective rather than curative effects; they were able to reduce both the incidence and pathogenicity of *C. gleosporoides* presumably due to multiple factors such as competition for space and nutrients, mycoparasitism and secretion of β-1, 3-glucanases, the latter probably attacking the phytopathogen´s cell wall [95].

In apples, *M. fructigena* is the causative agent of the brown rot. The fungus infects wounds and subsequently–and rapidly–develops the rather firm rot. The respective areas are arranged as small cottony masses forming pathogen characteristic concentric circles [96]. Five killer yeasts, isolated from the peel of apples, were tested in vivo and in vitro for their potential to tame the pathogen: *Schwanniomyces vanrijiae*, *Galactomyces geotrichum*, *Pichia kudriavzevii*, *D. hansenii* and *Rhodotorula glutinis* [97]. In vivo tests included three different approaches: (1) simultaneous application of both the antagonist and the pathogen, (2) placing the yeast 24 h prior to and (3) 24 h after infection. Disease incidence decreases of 84.02–89.5%, 80.1–86.9% and 56.3–86.9%, respectively, were obtained. In addition, it was shown that hydrolytic enzymes such as chitinases, pectinases, β-1, 3-glucanases, and proteases were produced.

More recently, *W. anomalus* BS91, *D. hansenii* K12a and *D. hansenii* AII4b were used to protect apples against *M. fructicola* [98]. All of the above strains were additionally and successfully tested against *Monilinia* spp. in stone fruits as well [89]. *W. anomalus* BS91 and *D. hansenii* K12a inhibited mycelial growth in vitro by 69 and 66%, respectively. *D. hansenii* AII4b was less effective (56%). Nevertheless, in vivo BS91 and K12a reduced the *M. fructicola* disease by 92 and 85%, respectively, while AII4b was less effective (70% disease reduction). Compared to untreated fruit, rot severity was reduced by approximately 52 up to 69%. Stress induction assays by measuring peroxidase and catalase levels in the peel of fruit indicated highest levels of peroxidase (POD) when wounds were treated with *W. anomalus* BS91. Lowest catalase (CAT) values were obtained with *D. hansenii* AII4b. The above findings led to the conclusion that the yeasts were able to modulate the activities of CAT and POD in apple tissues, leading the authors to suggest the yeasts promising candidates for developing effective biofungicides against *M. fructicola*.

Grapes are almost routinely infected by the rot-causing *A. carbonarius and A. niger*, both responsible for the accumulation of ochratoxin A, a potent nephrotoxic and carcinogenic compound that can even be found in secondary grape products [99,100].

Epiphytic yeasts isolated from grapes in Southern Italy, after molecular identification and inspection of the killer phenotype, were tested in vivo and in vitro to identify those representatives which are able to conquer the co-inoculated pathogenic fungi in their common ecological niches. *Issatchenkia orientalis* 17C2 and 16C2 efficiently reduced disease incidences of *A. carbonarius* and *A. niger* on grapes (100% growth reduction), due to the production of secondary antifungal metabolites and competition for essential nutrients [101].

Table 1 summarizes the known instances in which killer yeasts were used to combat or at least mitigate fungal diseases in fruit and crop.

## 5. Discussion and Conclusions

Since the discovery of the phenotype, the so-called killer yeasts became subjects of basic as well as applied research due to their sophisticated killing mechanisms and their potential applications in industry, medicine and agriculture. Regarding the latter, killer yeasts (and their toxins) have been used to control several economically important plant pathogens in a diversity of fruits, such as citrus, grape, papaya, stone fruit, tomato, and apple. They have shown their protective efficacy during both, pre- and postharvest stages.

The positive, fruit-protecting impact is apparently not merely due to a single action mechanism. Shelter from fungal infestation is probably brought about by accessory abilities, such as competition for space and nutrients and/or the production of antifungal compounds [28]. However, it is not to be excluded that killer proteins provide long-lasting effects improving their potential for biological control of crop-damaging organisms.

With respect to ecological and human health issues, one has to emphasize that many of the killer yeasts target specific fungal components, such as β-glucan, the main component of the cell wall. In addition, the majority of them cannot withstand the body temperature and a neutral pH-value) [44,86].

In spite of the numerous studies which have clearly demonstrated the advantages and benefits of using killer yeasts as biocontrol agents, commercial formulations are still not available. However, due to the obvious abundance and diversity of epiphytically thriving killer yeasts on fruit surfaces, and the continuous increase in the number of publications with respect to the search for novel killer strains as biocontrol agents, commercial formulations may soon be on hand.

## Figures and Tables

**Table 1:** Killer yeasts used as biological control agents in fruit (Figure 1).

Application Stage	Fruit	Pathogen	Antagonist Killer Yeast	Accessory Functions	References
**Preharvest**	Grapevine	*C. gloeosporioides*	*S. cerevisiae* GA8, CK and L24	Production of antifungal volatile compounds (*S. cerevisiae* GA8) and production of hydrolytic enzymes (β-1,3-glucanase and chitinase)	[77]
		*B. cinerea*	*P. membranifaciens* CYC 1106	N.D.	[78]
	Citrus	*C. acutatum*	*S. cerevisiae* ACB-AR1, ACB-KD1, ACB-CAT1, ACB-BG1, ACB-K1, and ACB-PE2	Competition for nutrients, inhibition of spore germination, production of hydrolytic enzymes (β-1,3-glucanase and chitinase) and production of antifungal volatile compounds (*S. cerevisiae* ACB-CAT1, ACB-BG1)	[79]
**Postharvest**	Lemon	*P. digitatum* and *P. italicum*	*P. fermentans* 27 and 28, *W. anomalus* 56, *C. lusitaniae* 146	Competition for nutrient and space	[39,82,83,84,85]
		*A. citri*	*D. hansenii* TEM8 and TEM17	N.D.	[90,91]
	Orange	*P. digitatum*	*W. anomalus* BS91 and BS92	Mycoparasitism, production of hydrolytic enzymes (β-1,3-glucanase)	[70,86,87]
			*C. lusitaniae* 146	Competition for nutrient and space	[85]
		*P. italicum*	*C. stellimalicola* ACBL-04, ACBL-05, ACBL-08 and *S. cereviciae* ACBL-11	Inhibition of spore germination, production of hydrolytic enzymes (chitinase)	[88]
	Mandarin	*P. digitatum*	*C. lusitaniae* 146	Competition for nutrient and space	[85]
	Grapefruit	*P. digitatum*	*C. lusitaniae* 146	Competition for nutrient and space	[85]
	Peach	*M. fructigena* and *M. fructicola*	*D. hansenii* KI2a and *W. anomalus* BS91	Production of hydrolytic enzymes (β-1,3-glucanase) and production of antifungal volatile compounds	[89]
	Plum	*M. fructigena* and *M. fructicola*	*D. hansenii* KI2a and *W. anomalus* BS91	Production of hydrolytic enzymes (β-1,3-glucanase) and production of antifungal volatile compounds (*W. anomalus* BS91)	[89]
	Tomato	*A. brassicicola* and *R. stolonifer*	*D. hansenii* TEM8 and TEM17	N.D.	[90,91]
	Papaya	*C. gloeosporioides*	*W. anomalus* 422 and *M. guilliermondii* 443	Competition for nutrients and space, mycoparasitism and production of hydrolytic enzymes (β-1,3-glucanase)	[94,95]
	Apple	*M. fructigena*	*S. vanrijiae*, *G. geotrichum*, *P. kudriavzevii*, *D. hansenii* and *R. glutinis*	Production of hydrolytic enzymes (chitinase, β-1,3-glucanase, pectinase, and protease)	[97]
		*M. fructicola*	*W. anomalus* BS91, *D. hansenii* 4II4b and KI2a	Induction of resistance plant	[98]
		*A. niger*	*D. hansenii* TEM8 and TEM17	N.D.	[90,91]
	Grape	*A. carbonarius* and *A. niger*	*I. orientalis* 2C2 and 16C2	Competition for nutrients	[101]

N.D.: not determined.
